# Maintained expression of genes associated with metabolism in the ventromedial hypothalamic nucleus despite development of leptin resistance during pregnancy in the rat

**DOI:** 10.1002/phy2.162

**Published:** 2013-11-19

**Authors:** Hollian R Phillipps, Sharon R Ladyman, David R Grattan

**Affiliations:** Centre for Neuroendocrinology and Department of Anatomy, School of Medical Sciences, University of OtagoDunedin, 9016, New Zealand

**Keywords:** BDNF, glucokinase, PACAP, pregnancy, ventromedial nucleus

## Abstract

Hyperphagia and weight gain to acquire energy stores for development and growth of the fetus and to prepare for the demands of lactation are important adaptations to support a healthy pregnancy. As a consequence, hypothalamic leptin resistance develops to enable maintenance of a positive energy state. During pregnancy there is a decrease in leptin receptor expression and reduced leptin-induced phospho signal transducer and activator of transcription 3 (pSTAT3) in the ventromedial nucleus of the hypothalamus (VMN), suggesting that the VMN is a key site of pregnancy-induced modification in the control of energy homeostasis. The aim of this study was to investigate expression levels of known gene targets, which are involved in metabolic regulation and glucosensing, within the VMN during pregnancy. Using in situ hybridization, pituitary adenylate cyclase–activated polypeptide (*Pacap*), brain-derived neurotrophic factor (*Bdnf*), and glucokinase messenger ribonucleic acid (mRNA) expression were localized in the hypothalamus of nonpregnant and day 14 pregnant rats, then expression levels were compared by quantitative polymerase chain reaction (qPCR) using laser capture microdissection of the VMN and arcuate nucleus. Despite significantly elevated plasma leptin and insulin concentrations, and lower blood glucose levels, during pregnancy, no significant changes in gene expression of *Pacap*, *Bdnf*, or glucokinase were detected between nonpregnant and day 14 pregnant groups. These data suggest that loss of leptin and insulin sensitivity in the VMN might allow gene expression to be maintained at normal/control levels in this nucleus, despite marked changes in the levels of these important regulatory hormones. These data provide further evidence for development of leptin resistance in the VMN as an adaptive response during pregnancy.

## Introduction

Pregnancy is a metabolically demanding state. Regulation of body weight and metabolism is modified to compensate for the increased energy demands associated with placental and fetal load, as well as generating a positive energy balance to allow fat deposition in preparation for lactation. In nonpregnant adults, body weight is homeostatically regulated by complex neuronal circuits that respond to numerous different factors, including peripheral hormones such as leptin and insulin (Morton et al. 2006; Belgardt and Bruning 2010). Leptin is an adipose-derived hormone that is secreted in proportion to the amount of body fat, and acts centrally to decrease appetite and increase metabolic rate. During pregnancy increases in fat mass are an important adaptation of the maternal body to prepare for the greatly increased metabolic demands of lactation. Leptin concentrations rise along with increases in fat mass, but food intake is also increased suggesting a state of leptin resistance. Moreover, exogenous leptin does not suppress food intake in pregnant rodents as it does in nonpregnant animals (Ladyman and Grattan 2004; Ladyman et al. 2012), confirming a pregnancy-induced loss of sensitivity to leptin. Pregnancy-induced leptin resistance is associated with a decrease in leptin receptor expression and a suppression of leptin-induced phospho signal transducer and activator of transcription 3 (pSTAT3) in the ventromedial nucleus of the hypothalamus (VMN) (Ladyman and Grattan 2004, 2005; Ladyman et al. 2012). These data suggest that the VMN may be a key site that undergoes adaptation to pregnancy to modify the regulation of energy balance. The aim of this study was to investigate genes within the VMN that have been implicated in the regulation of energy balance and thus may be altered during pregnancy and the associated state of positive energy balance.

Although the role of the VMN in the regulation of energy balance has been known for a long time, the specific neuronal phenotypes involved are not well understood. Early studies using lesions of the VMN demonstrated an anorectic role for this area (Anand and Brobeck 1951; Inoue and Bray 1977) and electrical stimulation of the VMN leads to a decrease in food intake (Ruffin and Nicolaidis 1999). The VMN contains both leptin and insulin receptors (Dhillon et al. 2006; Cotero and Routh 2009; Klockener et al. 2011), and isolated deletion of leptin receptors from this nucleus leads to obesity and increased food intake, indicating an important role of the VMN in the function of leptin (Dhillon et al. 2006). Recently, two factors expressed in neuronal populations within the VMN, pituitary adenylate cyclase–activating polypeptide (PACAP) and brain-derived neurotrophic factor (BDNF), have become a focus of work relating to the role of the VMN in energy balance. PACAP is a neuropeptide present throughout the central nervous system, but within the hypothalamus, it is most highly expressed in the VMN. A large proportion of leptin-responsive cells within the VMN contain PACAP, suggesting direct regulation of leptin on PACAP-containing neurons (Hawke et al. 2009). Fasted mice, and *ob/ob* mice, which lack functional leptin, both have reduced levels of VMN PACAP messenger ribonucleic acid (mRNA), and these levels can be restored by exogenous leptin administration (Hawke et al. 2009). Administration of PACAP directly into the brain leads to a suppression of food intake and increased energy expenditure (Morley et al. 1992; Chance et al. 1995; Mizuno et al. 1998; Hawke et al. 2009; Mounien et al. 2009). BDNF, a member of the neurotrophin family, has recently been demonstrated to play a distinct role in regulating energy balance (Vanevski and Xu 2013). BDNF is thought to act as an anorectic factor: like PACAP, infusions of BDNF into the lateral ventricles lead to a reduction in food intake and a number of BDNF mutant mouse models have demonstrated obesity phenotypes (Pelleymounter et al. 1995; Kernie et al. 2000; Rios et al. 2001). Furthermore, specific deletion of BDNF in the VMN and dorsomedial nucleus of the hypothalamus (DMN) of adult mice causes increased food consumption (Xu et al. 2003; Unger et al. 2007). Fasting leads to a reduction in *Bdnf* mRNA in the VMN. Leptin can induce *Bdnf* mRNA and protein expression in the VMN (Komori et al. 2006), although more recently, it has been shown that leptin receptors do not colocalize with BDNF in the VMN, indicating that this action of leptin on BDNF may be indirect (Liao et al. 2012). VMN BDNF-containing neurons express insulin receptors; thus, it is possible there is a direct action of insulin on these neurons (Liao et al. 2012). Given that both *Pacap* and *Bdnf* are expressed in the VMN and are known to be regulated by leptin and/or insulin, we hypothesized that pregnancy-induced leptin resistance within the VMN would result in a decrease in expression of these important VMN genes.

The VMN also contains glucose-sensing neurons that alter their firing rate in response to prevailing extracellular glucose concentrations (Oomura et al. 1969). Similar to its role in pancreatic beta-cells, glucokinase appears to act as a critical glucose sensor in a substantial proportion of these VMN neurons (Kang et al. 2006). Glucose-sensing neurons play a critical role in regulating release of counter-regulatory hormones, such as glucagon, in response to hypoglycemia (Borg et al. 1995, 1997; Levin et al. 2008). In situations such as diet-induced obesity, where there is defective central counter-regulatory response to hypoglycemia, VMN glucokinase mRNA expression is increased (Dunn-Meynell et al. 2002). The counter-regulatory response is also blunted during pregnancy, with a suppression of glucagon release (Rossi et al. 1993; Canniff et al. 2006) and an attenuated response of the autonomic system (Canniff et al. 2006). Therefore, we hypothesized that glucokinase mRNA would be increased during pregnancy, to contribute to pregnancy-induced attenuation in the counter-regulatory response to hypoglycemia.

Overall, the aim of the current study was to investigate genes within the VMN that have been implicated in energy homeostasis and thus may be altered during pregnancy and the associated state of positive energy balance. Initially, in situ hybridization was used to determine the hypothalamic localization of *Pacap*, *Bdnf*, and glucokinase mRNA in nonpregnant and day 14 pregnant rats, then laser capture microdissection (LMD) followed by quantitative polymerase chain reaction (qPCR) was used to assess expression levels of these genes in the two physiological states. Currently, there is only limited knowledge of metabolically relevant genes within the VMN and the target genes in this study represent three of the best characterized. Day 14 of pregnancy was selected as previous work has indicated that a state of leptin resistance in the VMN has developed, and food intake is significantly increased, by this stage of pregnancy (Ladyman and Grattan 2004).

## Material and Methods

### Animals

Female Sprague–Dawley rats, ranging in weight from 200–300 g and aged 10–14 weeks, were obtained from the colony at the University Of Otago. Animals were group housed in an environmentally controlled room with a 14 h light/10 h dark cycle, temperature maintained at 20 ± 1°C, and humidity in the range of 40–60%. All rats had free access to food and water. Experimental procedures involving rats were approved by the University of Otago Animal Ethics Committee in conjunction with the New Zealand Animal Welfare Act 1999 and Agricultural Compounds and Veterinary Medicines Act 1997. The estrous cycle was monitored by daily cytological examinations of vaginal smears. To generate timed pregnancies, proestrous females were housed overnight with a male Sprague–Dawley rat and presence of sperm in the vaginal smear the following morning was used as confirmation of pregnancy (day 0). For all experiments, nonpregnant rats were killed in the diestrous stage of the estrous cycle and pregnant rats on day 14 between 0900 and 1100 h. Nonpregnant (*n* = 7) and pregnant (*n* = 7) rats used for in situ hybridization histochemistry were given an intraperitoneal injection of sodium pentabarbitol (60 mg/mL, National Veterinary Supplies Ltd, New Zealand) to induce deep anesthesia prior to transcardial perfusion with 2% (w/v) paraformaldehyde in 0.1 mol/L phosphate buffer (PB). Brains were immediately removed and immersed in paraformaldehyde overnight, 30% (w/v) sucrose for 3–5 days, then rapidly frozen on dry ice and stored at −80°C until use. Tissue was collected from another group of nonpregnant (*n* = 11) and pregnant (*n* = 8) rats for LMD and qPCR analysis and hormone measurements. Following decapitation, rat brains were removed from the skull, and immediately frozen on dry ice before storage at −80°C until use. Trunk blood was collected at time of sacrifice and glucose level was immediately measured using a glucometer (Accu-Chek Performa by Roche, Mannheim, Germany). Blood samples were briefly centrifuged at 6000 *g* and plasma was collected and stored at −20°C. Plasma samples were analyzed using a rat leptin radioimmunoassay kit (Millipore, Billerica, MA) and a rat insulin radioimmunoassay kit (Millipore) according to manufacturer's instructions. Data for hormone and glucose concentrations were analyzed by one-way analyses of variance (ANOVA). The significant level was set at *P* < 0.05.

### In situ hybridization histochemistry

Coronal sections (16 *μ*m thickness) through the VMN and arcuate nucleus (Arc) were cut using a cryostat and float mounted onto Superfrost plus slides (Thermo Fisher Scientific NZ Ltd, North Shore City, New Zealand), air dried, and stored at −20°C until further use. [35-S]UTP-labeled riboprobes were synthesized using an in vitro transcription kit (Promega, Madison, WI) from purified DNA templates amplified by reverse transcription polymerase chain reaction (RT-PCR) using oligonucleotide primer sets for *Pacap* and glucokinase with SP6 and T7 RNA polymerase promoter sequences ligated to the 5′ ends of the forward and reverse primers, respectively (Table 1). A Digoxigenin (DIG) probe for *Bdnf* was developed using a DIG RNA labeling kit SP6/T7 (Roche, Manneheim, Germany) in accordance with manufacturer's instructions. Mini quick spin RNA columns (Roche, Indianapolis, IN) were used to remove unincorporated nucleotides or DIG and probes stored at −80°C for <7 days ([35-S]UTP probes) or <1 year (DIG probes) before use.

**Table 1:** Primer sets used for in situ hybridization histochemistry in the rat brain

Primer set	Sequence (5′–3′)	Amplicon	Accession number
*Pacap*	Forward CGGACTCAGCTTCCCTGGGA	230 bp	M63006
	Reverse GCAGGTACTTCCTGGCGGAC		F: 84-103
			R: 313-294
Glucokinase	Forward CACTCTGGGGCTTCGACCCT	202 bp	NM_012565
	Reverse CTTGAAGCTCGGGTGCAGCT		F: 1150–1169
			R: 1351–1332
*Bdnf*	Forward AGGACCAGAAGGTTCGGCCC	239 bp	NM_012513
	Reverse ATCTGCCGCTGTGACCCACT		F: 248–267
			R: 486–467

*Pacap*, pituitary adenylate cyclase–activated polypeptide; *Bdnf*, brain-derived neurotrophic factor.

For each target gene, six sections per animal (*n* = 7 for both nonpregnant and pregnant groups) were used for in situ hybridization. Sections were thawed at 55°C and postfixed in 2% (w/v) paraformaldehyde in 0.1 mol/L PB for 5 min. 0.5x saline sodium citrate (SSC, 75 mmol/L sodium chloride, 7.5 mmol/L trisodium citrate dihydrate) was used to wash sections prior to immersion in a proteinase k solution (2 *μ*g/mL, Roche, Manneheim, Germany) for 10 min to permeabilize tissue. Sections were washed in 0.5x SSC for 10 min, rinsed in 1x triethanolamine (TEA), and acetylation undertaken by agitation in 1x TEA containing 0.25% (v/v) acetic anhydride. Two changes of 2x SSC were used to wash sections prior to prehybridization. Sections were taken through an ethanol series (50, 80, 95, and 100%), immersed in chloroform for 5 min, then taken back through 100% (v/v) and 95% (v/v) ethanol, and air dried for <2 h. For hybridization [35-S]UTP probes were prepared by centrifuging at 12 000 *g* for 20 min at 4°C, removing the supernatant and washing with 70% (v/v) ethanol. Probes were spun for a further 5 min, the supernatant removed and resultant pellet air dried for 15–20 min before being dissolved in Tris-EDTA (TE) buffer. Both DIG (70 ng/section, *Bdnf*) and [35-S]UTP probes (6.0 × 10^5^ cpm per section, *Pacap* and 1.2 x 10^6^ cpm per section, Glucokinase) were mixed with tRNA (50 *μ*g/section), heated for 3 min at 95°C to denature, and combined with ice-cold hybridization buffer (50% [v/v] formamide, 0.3 mol/L Sodium chloride, 1x Denhardts solution, 20 mmol/L Tris, 5 mmol/L ethylenediaminetetraacetic acid [EDTA], 10% [w/v] dextran sulfate and 100 mmol/L Dithiothreitol [DTT]). Then 120 *μ*L of hybridization mixture was pipetted onto each slide, HybriSlips™ (Sigma-Aldrich, St Louis, MO) were applied and sections incubated at 55°C for 18 h.

Hybrislips were removed and sections washed twice in 2x SSC. For radioactive probes 2x SSC and 0.1x SSC also contained 10 mmol/L *β*-mercaptoethanol and 1 mmol/L EDTA. Sections were immersed in RNAse A (Sigma-Aldrich, 20 *μ*g/L in RNase buffer, 500 mmol/L NaCl, 10 mmol/L Tris) for 30 min. This was followed by two washes in 2x SSC for 10 min each, then a prolonged 2 h wash in 0.1x SSC for 2 h at 55°C (*Pacap* and *Bdnf*) or 65°C (Glucokinase). Sections were washed further in 0.5x SSC twice for 10 min. Slides containing sections used for *Pacap* and Glucokinase mRNA localization were taken through an ethanol series (50, 70, 95, and 100%), air dried overnight, and exposed to scientific imaging film (Kodak Biomax, MR film, Radiographic Supplies Ltd., Christchurch, New Zealand) for 10 days (*Pacap*) or 28 days (Glucokinase) to generate autoradiographs. Sections used for *Bdnf* DIG detection were washed further in two 5 min changes of buffer 1 (100 mmol/L Tris HCL, 150 mmol/L NaCl) prior to blocking with 2% (v/v) DIG block (Roche, Manneheim, Germany) in buffer 1 containing 1% (v/v) triton for 30 min. Incubation with DIG-Alkaline phosphatase Fab fragments (Roche, Manneheim, Germany) diluted 1:2000 in DIG blocking solution was undertaken for 48 h at 4°C.

Sections for *Bdnf* mRNA localization were washed twice in buffer 1 for 10 min, once in buffer 3 (100 mmol/L Tris-HCL, 100 mmol/L NaCl and 50 mmol/L MgCl_2_) for 10 min and alkaline phosphatase activity present on sections was detected by incubation with a 4-nitro blue tetrazolium chloride/5-bromo-4-chloro-3-indolyl phosphate, toluidine salt (NBT/BCIP) solution for 4 h at room temperature. Four changes in 1x phosphate/saline/EDTA (SSPE) buffer lasting 30 min each washed sections thoroughly prior to immersion in distilled water and an ethanol series (70, 95, and 100%). Sections for *Pacap* and Glucokinase mRNA localization were dipped in Ilford K.5D nuclear emulsion (Harman Technology Limited, Cheshire, England) and stored at 4°C for 2 (*Pacap*) or 12 (Glucokinase) weeks. Kodak D19 developer and Ilford rapid fixer were used to develop sections. All sections were dried at 42°C for 1 h, immersed in two changes of Xylene, and coverslipped using vectamount (Vector Laboratories, Inc, Burlingame, CA). Sections were viewed using an Olympus AX70 Provis light microscope (Olympus Optical Co. Ltd., Tokyo, Japan) and images captured with a Spot RT digital camera and associated software. Positive hybridization was assessed using Image J 1.43u software (National Institutes of Health, Bethesda, MD) and defined as an mRNA signal >3 times background. Brightness, contrast or correction to color balance in images were undertaken using Adobe Photoshop CS5 extended version 12.0.4 x64 (Adobe Systems Incorporated, San Jose, CA).

### Laser capture microdissection

**Figure 1:**
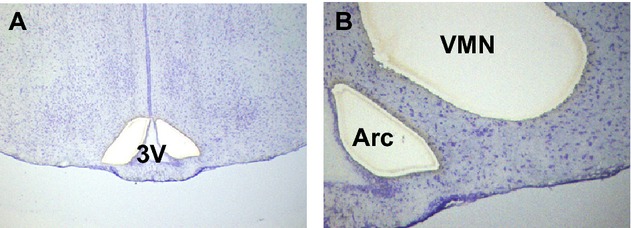
Laser capture microdissection of the VMN and arcuate nucleus (Arc) from coronal brain sections collected from nonpregnant rats and day 14 pregnant rats. (A, B) Part of a thionin-stained coronal rat brain section used for LMD. (A) Two holes where the Arc has been dissected out on both sides of the third ventricle (3V). The VMN is still intact. (B) Holes where the Arc and VMN have been dissected out on the left side of the third ventricle shown in A. VMN, ventromedial nucleus of the hypothalamus; LMD, laser capture microdissection.

Coronal cryosections (10 *μ*m thickness) taken at 40 *μ*m intervals through the VMN and Arc were cut and thaw mounted onto Leica polyethylene naphthalate (PEN) membrane slides (Bio-strategy, Auckland, New Zealand) and stored at −80°C for up to 30 h. Sections were postfixed in 70% (v/v) ethanol and rapidly stained with 0.1% (v/v) thionin staining solution. A Leica LMD system (Leica, Wetzlar, Germany) was used to isolate tissue from the VMN and Arc of the hypothalamus (Fig. 1). Tissue was collected from 16 VMN sections from each animal (*n* = 6 for both nonpregnant and pregnant groups). Dissected tissue was collected and placed into lysis buffer within 1 h (Qiagen RNeasy Micro Kit, Valencia, CA) containing 1% (v/v) *β*-mercaptoethanol and total RNA extracted in accordance with manufacturer's instructions using the Qiagen RNeasy Micro Kit including an on-column DNAse digestion. Resultant RNA quality and quantity was assessed by running an Agilent small RNA assay (Agilent Technologies, Inc, Waldbronn, Germany) according to manufacturer's instructions in an Agilent 2100 Bioanalyzer. Only samples with intact 18S and 28S RNA peaks and RIN values >6.00 were included in subsequent qPCR.

### Quantitative polymerase chain reaction

First-strand cDNA was produced from 1–2 ng total RNA with random primers using Superscript™ III Reverse transcriptase (Invitrogen New Zealand Limited, Victoria, Australia) according to the manufacturer's protocol. An RT negative control was included for each sample. The resultant cDNA was stored at −20°C prior to qPCR. Oligonucleotide primer and probe sequences specific for *Bdnf*, Glucokinase and *Pacap* were designed using PrimerQuest^SM^ software (Integrated DNA Technologies Inc., Singapore Science Park II, Singapore) (Table 2). *β*-actin was used as a reference gene and all probes (Integrated DNA Technologies Inc.) contained a 5′ 6-FAM reporter dye and both internal ZEN™ and 3′ Iowa black FQ quenchers for increased sensitivity (Table 2).

**Table 2:** Quantitative PCR primer/probe sets used for identification of mRNA transcripts in laser capture microdissected VMN and arcuate nucleus samples in diestrous rats and day 14 pregnant rats

Primer set	Sequence (5′–3′)	Amplicon	Accession number	Efficiencies
*Pacap*	Forward GGAAACCCGCTGCAAGACTTCTAC	150 bp	M63006	1.02
	Reverse CAAGACTTTGCGGTAGGCTTCGTT		F 136-159	
	Probe ACGCCTACGCCCTTTACTACCCAGCCGACA		R 285-262	
			P 206-225	
Glucokinase	Forward CGAGCTTCAAGGAGCGGTTTCAC	81 bp	NM_012565	1.02
	Reverse CCTCCTCTGATTCGATGAAGGTGATTTCG		F: 1341-1363	
	Probe AGTGTGCGCAGGCTGACACCCAACT		R: 1421-1393	
			P: 1367-1391	
*Bdnf*	Forward AGAAGGTTCGGCCCAACGAAGAAA	103 bp	NM_012513	0.94
	Reverse AGAAAGAGCAGAGGAGGCTCCAA		F: 890-913	
	Probe TCCCGGGTGATGCTCAGCAGTCAAGTGCCT		R: 992-969	
			P: 940-968	
*β*-actin	Forward AGATGACCCAGATCATGTTTGAGA	86 bp	NM_031144	0.95
	Reverse ACCAGAGGCATACAGGGACAA		F: 434-457	
	Probe TCAACACCCCAGCCATGTACGTAGCC		R: 519-499	
			P: 461-486	

PCR, polymerase chain reaction; VMN, ventromedial nucleus of the hypothalamus; *Pacap*, pituitary adenylate cyclase–activated polypeptide; *Bdnf*, brain-derived neurotrophic factor.

Quantitative PCR was undertaken by set up of a reaction mix containing optimized final concentrations of primers (600 nmol/L, all genes) and probes (200 nmol/L [*β-actin*, *Pacap*, and glucokinase] 150 nmol/L [*Bdnf*] [Table 2]), LightCycler ® 480 Probes Master (1x concentration, Roche Applied Science, Mannheim, Germany) and RNase free water. This was combined with template cDNA (2 *μ*L, undiluted). Samples were set up in duplicate as singleplex reactions in white LightCycler® 480 96-well plates (Roche) to give a final 20-*μ*L reaction volume. All plates contained negative controls, in which template cDNA was replaced with RNAse free water for each gene reaction mix. A set of calibrator LMD samples obtained from either the Arc or VMN from both the control and day 14 pregnant groups were also included on each plate.

The LightCycler® 480 Instrument II Real-time PCR system (Roche) was used to detect resultant fluorescent signal for each gene. Each plate was run in the machine using a thermocycling protocol consisting of an initial preincubation step at 95°C for 10 min to increase detection sensitivity, then 45 amplification cycles starting at 95°C for 10 sec, 60°C for 30 sec, and 72°C for 1 sec, followed by a single cooling step at 40°C for 10 sec. The resulting data from each plate were run through an absolute quantitation/second derivative maximum analysis using the LightCycler® 480 software 1.5.0.39 to obtain quantification cycle (Cq) values for each transcript and these data were analyzed using the comparative Cq method. The Cq value is defined as the cycle number in which the fluorescent signal (proportional to the amount of amplified product) for each reaction is first detected as significantly above background fluorescent levels (Bustin 2000). mRNA transcripts with a Cq value ≥35 were considered below the level of detection and excluded from subsequent analysis. In brief, the difference between the Cq value obtained for *β*-actin and either *Bdnf*, *Pacap* or glucokinase in each sample is obtained to give the ΔCq. Since this value does not account for a normalizer a ΔΔCq value is obtained by averaging the ΔCq values for *β*-actin and determining the difference between the mean ΔCq for *β*-actin and the gene of interest ΔCq for each animal. The presence of a fold difference in expression, if any, is then determined using the equation 2^−ΔΔCq^. A Student's *t*-test using ΔCq values obtained from each animal set was used to evaluate the presence of any significant differences in relative gene expression between the two groups. Data are presented as relative gene expression with comparison to the diestrous control group ±SEM and a *P*-value <0.05 was considered statistically significant.

## Results

**Figure 2:**
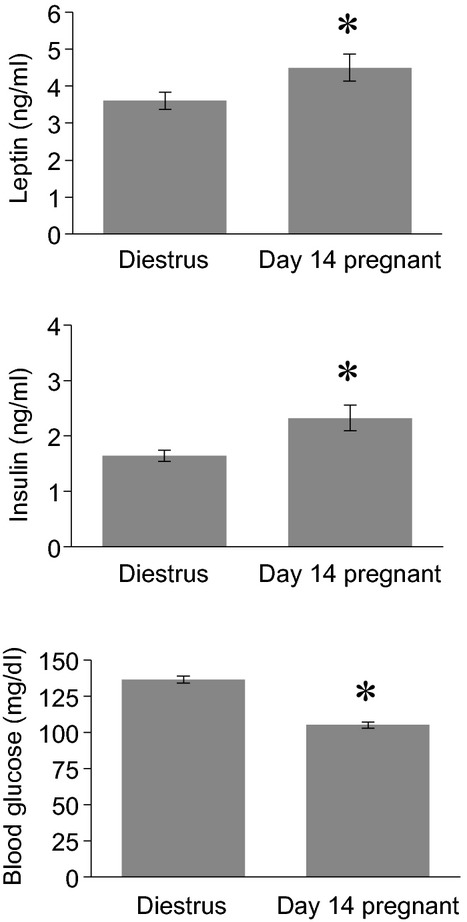
Leptin (A), insulin (B), and blood glucose (C) levels in nonfasting nonpregnant rats (*n* = 11) in the diestrous phase of the estrous cycle and day 14 pregnant rats (*n* = 8). Data presented as mean ± SEM. *Significantly different to nonpregnant group (*P* < 0.05).

Serum leptin and insulin concentrations were significantly elevated on day 14 of pregnancy (*n* = 8) compared to levels in nonpregnant rats (*n* = 11) (one-way ANOVA) (Fig. 2). Blood glucose level was significantly lower on day 14 of pregnancy (*n* = 11) compared to the nonpregnant group (*n* = 8) (one-way ANOVA) (Fig. 2). Despite the increases in these anorectic hormones, food intake is markedly increased at this time during pregnancy, indicative of the development of leptin resistance during pregnancy (Ladyman and Grattan 2004; Ladyman 2008).

**Figure 3:**
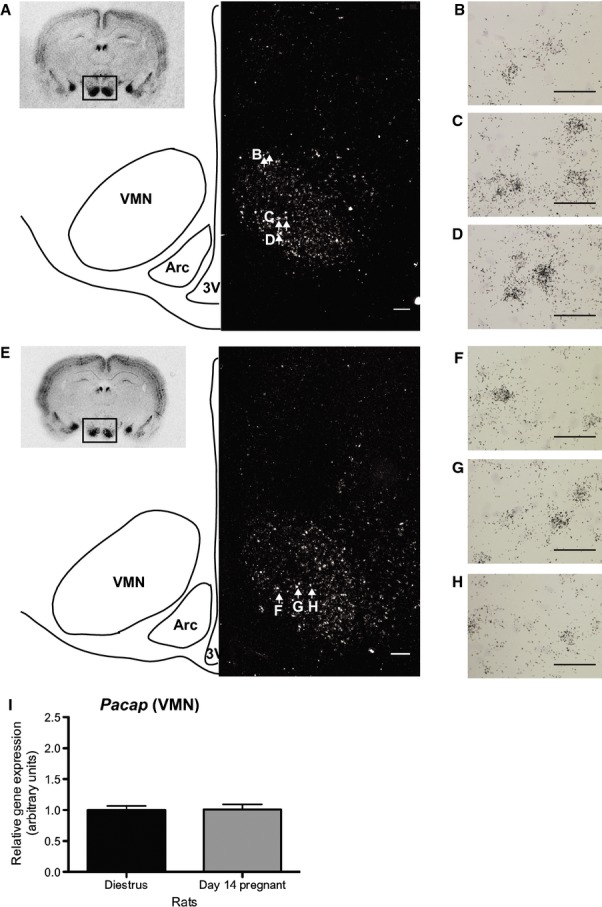
*Pacap* mRNA expression in the VMN of the rat hypothalamus. (A) Diagram consisting of an autoradiographic image of a coronal brain section (insert, top right) from a nonpregnant rat showing high positive hybridization for *Pacap* mRNA in the VMN. The boxed area is shown below in the line diagram and dark-field image. The line diagram shows the approximate location of the VMN and Arc in the corresponding dark-field image. A positive signal for *Pacap* mRNA is present in the VMN on the dark-field image. (B–D) High-powered bright-field images showing silver grain clusters representative of *Pacap* -positive cells in three different locations in the VMN (white arrows in A correspond to the location of images B–D). (E) Same diagram setup as in A but in a day 14 pregnant rat. The VMN of the pregnant rat, similar to nonpregnant rat, shows a positive signal for *Pacap* mRNA. (F–H) High-powered bright-field images showing silver grain clusters indicative of *Pacap* mRNA expression at three different locations in the VMN (white arrows in E indicate the locations of images F–H). Minimal to no positive *Pacap* mRNA expression was detected in the Arc. 3V indicates the third ventricle. Scale bars A and E 200 *μ*m, B–D and F–G 20 *μ*m. (I) Graph showing no change in *Pacap* mRNA levels in laser capture microdissected VMN samples from nonpregnant rats (*n* = 6) and day 14 pregnant rats (*n* = 6). Data in I presented as relative gene expression with comparison to control group ± SEM. VMN, ventromedial nucleus of the hypothalamus; *Pacap*, pituitary adenylate cyclase–activated polypeptide.

Pituitary adenylate cyclase–activated polypeptide mRNA expression within the hypothalamus was predominantly confined to the VMN (Fig. 3). Distinct clusters of high positive hybridization for *Pacap* mRNA were detected throughout the VMN (Fig. 3). A few *Pacap*-positive cells were observed on the ventrolateral border of the rostal Arc, but quantitation of relative gene expression in the Arc was not possible as levels were negligible when analyzed by qPCR, once the whole nucleus was included in the analysis (data not shown). In the VMN, there was no difference in expression levels of *Pacap* mRNA in the pregnant group, compared with the nonpregnant controls (Student's *t-*test, *n* = 6 per group) (Fig. 3).

**Figure 4:**
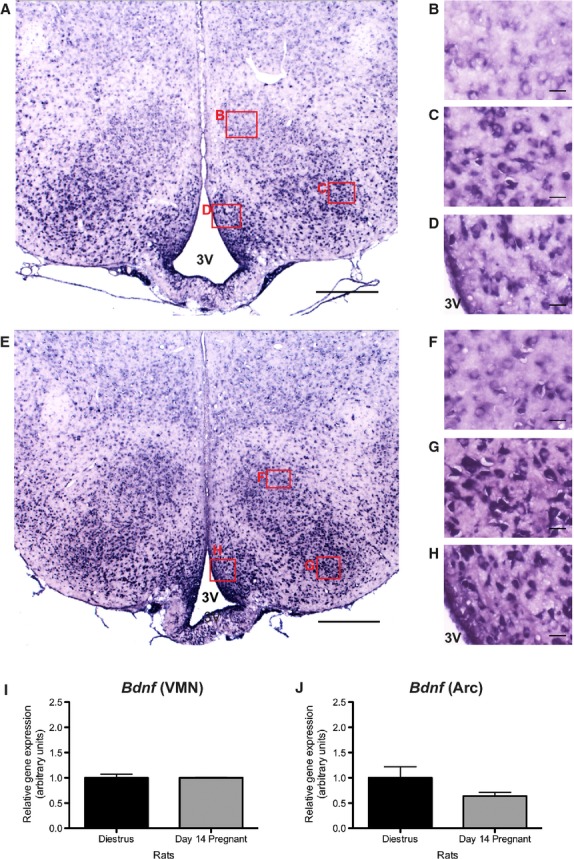
*Bdnf* mRNA expression in the rat ventromedial and arcuate nuclei. (A) Low-powered image of the medial basal hypothalamus from a nonpregnant rat showing positive *Bdnf* mRNA expression in the VMN and Arc. (B) Image of the dorsal medial aspect of the VMN. A low positive signal for *Bdnf* mRNA is apparent in some cells. (C and D) High-powered images of cells in the dorsal lateral portion of the VMN (C) and Arc (D). Intense positive hybridization for *Bdnf* mRNA is widespread. (E) Low-powered image of the medial basal hypothalamus from a day 14 pregnant rat showing positive hybridization for *Bdnf* mRNA in both the VMN and Arc. (F) High-powered image of the dorsal medial VMN showing low *Bdnf* mRNA expression. (G and H) High positive *Bdnf* mRNA expression is evident in the ventral lateral VMN (G) and Arc (H). Red boxes in A and E indicate the location of images B–D and F–H, respectively. 3V indicates the third ventricle. Scale bars A and E 200 *μ*m, B–D and F–H 25 *μ*m. (I and J) Graphs showing no significant change in levels of *Bdnf* mRNA in both the VMN and Arc from diestrous (*n* = 6) and day 14 pregnant rats (*n* = 6). Data in I and J presented as relative gene expression with comparison to control group ± SEM. *Bdnf*, brain-derived neurotrophic factor; VMN, ventromedial nucleus of the hypothalamus.

Widespread *Bdnf* mRNA expression was evident in the mediobasal hypothalamus, particularly within the Arc (Fig. 4). In the VMN, positive hybridization was predominantly localized to the ventrolateral aspect of the nucleus, with minimal mRNA expression observed in the dorsomedial portion that is more typically associated with leptin-responsive neurons (Fig. 4). Pregnancy did not affect the pattern of *Bdnf* mRNA expression, nor the levels of mRNA of *Bdnf* measured in either the ventromedial or arcuate nuclei when measured by qPCR (Student's *t-*test, *n* = 6 per group) (Fig. 4).

**Figure 5:**
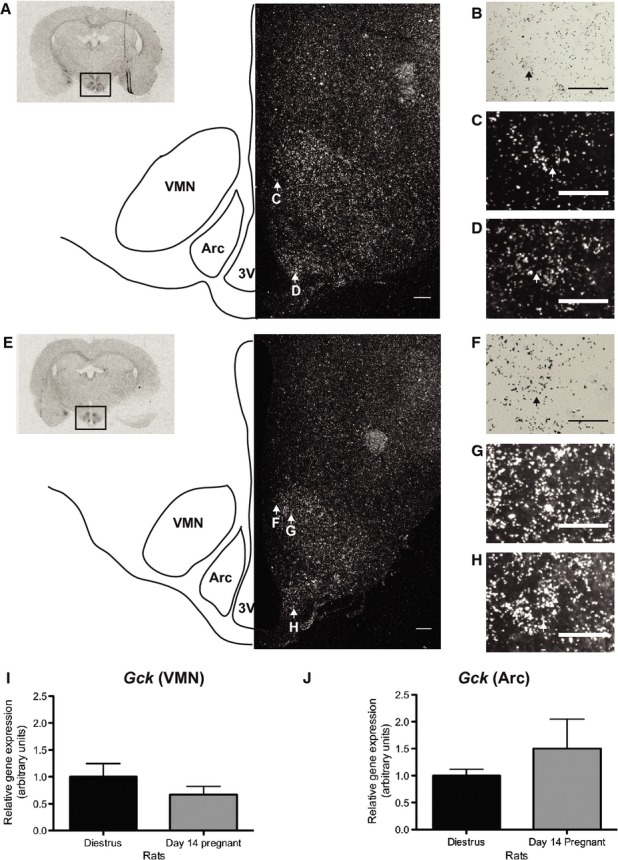
Glucokinase mRNA expression in the rat medial basal hypothalamus. (A) Composite diagram containing an insert (top left corner) depicting an autoradiographic image showing positive hybridization for glucokinase mRNA in the hypothalamus of a nonpregnant rat. The boxed area is shown in a larger form below as a combination of a line diagrammatic representation and dark-field image. The line diagram depicts the approximate locations of the VMN and arcuate nucleus (Arc) in the dark-field image. Low positive expression for glucokinase mRNA is present in both the Arc and VMN. (B) High-powered bright-field image of the VMN. An example of a glucokinase-positive cell is indicated by the black arrow. (C and D) High-powered dark-field images of glucokinase-positive cells (indicated by white arrows) in the VMN and Arc, respectively. The corresponding cells are indicated by white arrows in A. (E) The same type of composite diagram as in A but from a day 14 pregnant rat. A low positive signal for glucokinase mRNA is detectable in both the Arc and VMN as shown in the dark-field image. (F) High-powered bright-field image showing an example of glucokinase mRNA positive cells (indicated by black arrow) in the VMN. (G and H) Dark-field examples of glucokinase mRNA positive cells (indicated by white arrows) in the VMN and Arc, respectively. The location of these corresponding cells are indicated by white arrows in E. 3V indicates the third ventricle. Scale bars A and E 200 *μ*m, B and F 20 *μ*m, C, D, G, H 25 *μ*m. Graphs I and J show relative levels of Glucokinase mRNA expression in laser capture microdissected VMN (I) and arcuate (J) nucleus samples obtained from nonpregnant (*n* = 6) and day 14 pregnant (*n* = 6) rats. Data in I and J presented as relative gene expression with comparison to control group ± SEM. VMN, ventromedial nucleus of the hypothalamus.

Glucokinase mRNA expression was diffuse throughout both the VMN and Arc (Fig. 5). Furthermore, expression levels appeared uniform within both the dorsomedial and ventrolateral aspects of the VMN, and no differences were detected between nonpregnant and day 14 pregnant rats (Fig. 5). This was confirmed by the absence of any significant change in glucokinase mRNA expression levels, as measured by qPCR, in the VMN and Arc samples obtained from nonpregnant and day 14 pregnant rats (Student's *t-*test, *n* = 6 per group) (Fig. 5).

No positive signals were detected on any sections used for in situ hybridization histochemistry treated with sense probes for Glucokinase and *Pacap* or a DIG sense probe for *Bdnf* mRNA. Moreover, no positive expression was evident in RT negative or water control wells in qPCR experiments.

## Discussion

Alterations to food intake and metabolism are important adaptive responses during pregnancy to meet the constant energy demands of the fetus and to allow for the acquisition of maternal fat stores for subsequent use during lactation. To facilitate this state of positive energy balance, leptin resistance develops during pregnancy allowing increased food intake to be maintained in the face of rising levels of leptin (Ladyman et al. 2010). There are at least three distinct mechanisms of leptin resistance during pregnancy in rodents: (1) Arc insensitivity, as demonstrated by a lack of change of neuropeptide Y (NPY), agouti-related peptide (AgRP), and proopiomelanocortin (POMC) mRNA in response to the increased leptin observed during pregnancy (Ladyman et al. 2009); (2) alpha-MSH insensitivity, as demonstrated by a lack of satiety response to alpha-MSH administration (Ladyman et al. 2009); and (3) VMN insensitivity, as demonstrated by decrease leptin receptor expression and impaired leptin-induced pSTAT3 in this nucleus during pregnancy (Ladyman and Grattan 2004, 2005; Ladyman et al. 2012).This study sought to further investigate this third mechanism, by evaluating changes in genes known to be involved in energy homeostasis within the VMN. Although the VMN has long been known to be involved in the regulation of energy balance, the specific neuronal phenotype of the neurons involved is only just beginning to be understood. Here, we examined three genes on day 14 of pregnancy that may be candidates to mediate actions of metabolic hormones in the VMN: *Pacap*, *Bdnf* and glucokinase. Surprisingly, despite significant changes in leptin and insulin secretion during pregnancy, and reduced levels of plasma glucose, expression for these genes did not change during pregnancy. These data are consistent with a loss of sensitivity to leptin and insulin in the VMN during pregnancy.

Pituitary adenylate cyclase–activated polypeptide-containing neurons in the VMN are responsive to leptin, and may act as a downstream pathway through which leptin induces satiety (Hawke et al. 2009). Despite the increased leptin concentrations of pregnancy, however, *Pacap* mRNA levels in the VMN were not increased. Although we did not see a decrease in expression, as originally hypothesized, the absence of any change in expression in response to elevated leptin is consistent with previous evidence showing decreased leptin receptor expression and impaired leptin-induced pSTAT3 in this nucleus during gestation (Ladyman and Grattan 2004, 2005), indicative of decreased sensitivity to leptin in PACAP neurons during pregnancy. Such a loss of sensitivity may provide an adaptative mechanism during pregnancy, to minimize the potential anorectic effects of PACAP on food intake, and possibly the PACAP mediated stimulation of energy expenditure, to support the pregnancy-induced state of positive energy balance.

BDNF is a member of the neurotrophin family and regulates neuronal development and synaptic plasticity. Recently, it has also been demonstrated to play a distinct role in regulating energy balance (Vanevski and Xu 2013). It is highly expressed in the hypothalamus, where it acts as a satiety factor (Pelleymounter et al. 1995; Kernie et al. 2000; Rios et al. 2001), particularly in the VMN and DMN (Xu et al. 2003; Unger et al. 2007). While it is now thought that leptin does not act directly on *Bdnf*-containing neurons within the VMN (Liao et al. 2012), it does appear to indirectly stimulate *Bdnf* mRNA through the melanocortin system (Xu et al. 2003). In the current study, despite elevated leptin levels during pregnancy, there were no changes in the levels of *Bdnf* mRNA in the VMN. It seems possible that this lack of response could be mediated by a change in response to melanocortin signaling. Unlike diet-induced or age-induced leptin resistance, which are accompanied by normal responsiveness, or in some cases enhanced responsiveness, to alpha-MSH (Pierroz et al. 2002; Zhang et al. 2004), pregnancy-induced leptin resistance is associated with a loss of response to alpha-MSH, suggesting that impaired melanocortin signaling may underlie at least part of the lack of response to leptin (Ladyman et al. 2009). BDNF-containing neurons in the VMN express insulin receptors (Liao et al. 2012), and insulin acts with leptin to regulate the translation of long 3′ UTR *Bdnf* mRNA in dendrites. It is this form of *Bdnf* mRNA that is likely to be involved in BDNF-mediated suppression of feeding (Liao et al. 2012).The absence of a change in *Bdnf* mRNA during pregnancy, despite significantly elevated insulin, suggests that pregnancy may also be associated with insulin resistance in the hypothalamus.

In this study, we also showed that there is no change in glucokinase mRNA in the VMN during pregnancy. This is interesting, as pregnancy is associated with impaired counter-regulatory response to hypoglycemia (Rossi et al. 1993; Canniff et al. 2006). In other situations where counter-regulatory responses are impaired, such as diet-induced obesity, elevated glucokinase expression is observed (Dunn-Meynell et al. 2002). This suggests that the change in counter-regulatory response during pregnancy is mediated through different mechanisms, and is unlikely to involve glucokinase. It should be noted that as opposed to pathological changes in obesity, the blunted counter-regulatory response during pregnancy is adaptive, altering maternal glucose homeostasis to provide the optimal environment for the growing fetus. An important caveat is that in the current study only glucokinase mRNA was measured, and not activity of the enzyme. Leptin has been shown to decrease VMN glucokinase enzyme activity in rat hypothalamic slice explants (Sanz et al. 2007). In a neuronal cell line (GT1 cells), leptin does not affect glucokinase mRNA expression (Sanz et al. 2007), therefore it is possible that impaired leptin signaling in the VMN during pregnancy (Ladyman and Grattan 2004, 2005) may result in increased glucokinase activity without changes in glucokinase mRNA levels.

Pregnancy is associated with hyperphagia and increased fat deposition, elevated leptin and insulin secretion, and a loss of response to the anorectic effects of leptin. In this study, we sought to evaluate the consequences of pregnancy-induced leptin resistance in the VMN on three genes that are preferentially expressed in this nucleus and are known to be involved in regulation of glucose and body weight homeostasis. Although these genes did not appear to be differentially regulated during pregnancy, the absence of change in the face of significantly altered leptin and insulin concentrations is indicative of a loss of sensitivity to these important metabolic hormones. It is unlikely that changes in these genes in the VMN can account for the hyperphagia of pregnancy, as levels were not different from the nonpregnant state. It seems likely that hyperphagia may be mediated by other hormones that are elevated during pregnancy and that can stimulate appetite such as prolactin or progesterone (Hervey and Hervey 1967; Gerardo-Gettens et al. 1989a,b; Sauve and Woodside 1996). Nevertheless, the apparent loss of sensitivity to leptin and insulin may be important to prevent the normal anorectic consequences of elevations in these hormones, allowing increased food intake to be maintained during pregnancy, unopposed by the regulatory feedback response that would normally be generated by increasing fat mass and rising leptin levels. This is an adaptive response, essentially buffering the hypothalamus from the consequences of high leptin, when the satiety actions of leptin are not desirable. This is analogous to how skeletal muscle and fat tissue becomes insulin resistant during pregnancy, to facilitate glucose transport to the fetus (Leturque et al. 1984; Ryan et al. 1985; Hauguel et al. 1987; Holness et al. 1991).

The hormonal changes associated with pregnancy mediate a number of adaptive responses to facilitate a positive energy balance in preparation for subsequent metabolic demands of lactation. Hypothalamic leptin resistance is a key mechanism, particularly within the VMN, alleviating restrictions on food intake and allowing pregnancy-specific orexigenic signals to drive food intake and accumulation of fat stores. The results from the current study have shown that despite changes in concentrations of metabolic signals such as leptin, insulin, and glucose, known VMN genes involved in the regulation of energy balance do not differ from levels in the nonpregnant state. These data provide further evidence for development of leptin resistance in the VMN as an adaptive response during pregnancy.

## References

[b1] Anand BK, Brobeck JR (1951). Hypothalamic control of food intake in rats and cats. Yale J. Biol. Med.

[b2] Belgardt BF, Bruning JC (2010). CNS leptin and insulin action in the control of energy homeostasis. Ann. N. Y. Acad. Sci.

[b3] Borg WP, Sherwin RS, During MJ, Borg MA, Shulman GI (1995). Local ventromedial hypothalamus glucopenia triggers counterregulatory hormone release. Diabetes.

[b4] Borg MA, Sherwin RS, Borg WP, Tamborlane WV, Shulman GI (1997). Local ventromedial hypothalamus glucose perfusion blocks counterregulation during systemic hypoglycemia in awake rats. J. Clin. Invest.

[b5] Bustin SA (2000). Absolute quantification of mRNA using real-time reverse transcription polymerase chain reaction assays. J. Mol. Endocrinol.

[b6] Canniff KM, Smith MS, Lacy DB, Williams PE, Moore MC (2006). Glucagon secretion and autonomic signaling during hypoglycemia in late pregnancy. Am. J. Physiol. Regul. Integr. Comp. Physiol.

[b7] Chance WT, Thompson H, Thomas I, Fischer JE (1995). Anorectic and neurochemical effects of pituitary adenylate cyclase activating polypeptide in rats. Peptides.

[b8] Cotero VE, Routh VH (2009). Insulin blunts the response of glucose-excited neurons in the ventrolateral-ventromedial hypothalamic nucleus to decreased glucose. Am. J. Physiol. Endocrinol. Metab.

[b9] Dhillon H, Zigman JM, Ye C, Lee CE, McGovern RA, Tang V (2006). Leptin directly activates SF1 neurons in the VMH, and this action by leptin is required for normal body-weight homeostasis. Neuron.

[b10] Dunn-Meynell AA, Routh VH, Kang L, Gaspers L, Levin BE (2002). Glucokinase is the likely mediator of glucosensing in both glucose-excited and glucose-inhibited central neurons. Diabetes.

[b11] Gerardo-Gettens T, Moore BJ, Stern JS, Horwitz BA (1989a). Prolactin stimulates food intake in a dose-dependent manner. Am. J. Physiol.

[b12] Gerardo-Gettens T, Moore BJ, Stern JS, Horwitz BA (1989b). Prolactin stimulates food intake in the absence of ovarian progesterone. Am. J. Physiol.

[b13] Hauguel S, Gilbert M, Girard J (1987). Pregnancy-induced insulin resistance in liver and skeletal muscles of the conscious rabbit. Am. J. Physiol.

[b14] Hawke Z, Ivanov TR, Bechtold DA, Dhillon H, Lowell BB, Luckman SM (2009). PACAP neurons in the hypothalamic ventromedial nucleus are targets of central leptin signaling. J. Neurosci.

[b15] Hervey E, Hervey GR (1967). The effects of progesterone on body weight and composition in the rat. J. Endocrinol.

[b16] Holness MJ, Changani KK, Sugden MC (1991). Progressive suppression of muscle glucose utilization during pregnancy. Biochem. J.

[b17] Inoue S, Bray GA (1977). The effects of subdiaphragmatic vagotomy in rats with ventromedial hypothalamic obesity. Endocrinology.

[b18] Kang L, Dunn-Meynell AA, Routh VH, Gaspers LD, Nagata Y, Nishimura T (2006). Glucokinase is a critical regulator of ventromedial hypothalamic neuronal glucosensing. Diabetes.

[b19] Kernie SG, Liebl DJ, Parada LF (2000). BDNF regulates eating behavior and locomotor activity in mice. EMBO J.

[b20] Klockener T, Hess S, Belgardt BF, Paeger L, Verhagen LA, Husch A (2011). High-fat feeding promotes obesity via insulin receptor/PI3K-dependent inhibition of SF-1 VMH neurons. Nat. Neurosci.

[b21] Komori T, Morikawa Y, Nanjo K, Senba E (2006). Induction of brain-derived neurotrophic factor by leptin in the ventromedial hypothalamus. Neuroscience.

[b22] Ladyman SR (2008). Leptin resistance during pregnancy in the rat. J. Neuroendocrinol.

[b23] Ladyman SR, Grattan DR (2004). Region-specific reduction in leptin-induced phosphorylation of signal transducer and activator of transcription-3 (STAT3) in the rat hypothalamus is associated with leptin resistance during pregnancy. Endocrinology.

[b24] Ladyman SR, Grattan DR (2005). Suppression of leptin receptor messenger ribonucleic acid and leptin responsiveness in the ventromedial nucleus of the hypothalamus during pregnancy in the rat. Endocrinology.

[b25] Ladyman SR, Tups A, Augustine RA, Swahn-Azavedo A, Kokay IC, Grattan DR (2009). Loss of hypothalamic response to leptin during pregnancy associated with development of melanocortin resistance. J. Neuroendocrinol.

[b26] Ladyman SR, Augustine RA, Grattan DR (2010). Hormone interactions regulating energy balance during pregnancy. J. Neuroendocrinol.

[b27] Ladyman SR, Fieldwick DM, Grattan DR (2012). Suppression of leptin-induced hypothalamic JAK/STAT signalling and feeding response during pregnancy in the mouse. Reproduction.

[b28] Leturque A, Burnol AF, Ferre P, Girard J (1984). Pregnancy-induced insulin resistance in the rat: assessment by glucose clamp technique. Am. J. Physiol.

[b29] Levin BE, Becker TC, Eiki J, Zhang BB, Dunn-Meynell AA (2008). Ventromedial hypothalamic glucokinase is an important mediator of the counterregulatory response to insulin-induced hypoglycemia. Diabetes.

[b30] Liao GY, An JJ, Gharami K, Waterhouse EG, Vanevski F, Jones KR (2012). Dendritically targeted Bdnf mRNA is essential for energy balance and response to leptin. Nat. Med.

[b31] Mizuno Y, Kondo K, Terashima Y, Arima H, Murase T, Oiso Y (1998). Anorectic effect of pituitary adenylate cyclase activating polypeptide (PACAP) in rats: lack of evidence for involvement of hypothalamic neuropeptide gene expression. J. Neuroendocrinol.

[b32] Morley JE, Horowitz M, Morley PM, Flood JF (1992). Pituitary adenylate cyclase activating polypeptide (PACAP) reduces food intake in mice. Peptides.

[b33] Morton GJ, Cummings DE, Baskin DG, Barsh GS, Schwartz MW (2006). Central nervous system control of food intake and body weight. Nature.

[b34] Mounien L, Do Rego JC, Bizet P, Boutelet I, Gourcerol G, Fournier A (2009). Pituitary adenylate cyclase-activating polypeptide inhibits food intake in mice through activation of the hypothalamic melanocortin system. Neuropsychopharmacology.

[b35] Oomura Y, Ono T, Ooyama H, Wayner MJ (1969). Glucose and osmosensitive neurones of the rat hypothalamus. Nature.

[b36] Pelleymounter MA, Cullen MJ, Wellman CL (1995). Characteristics of BDNF-induced weight loss. Exp. Neurol.

[b37] Pierroz DD, Ziotopoulou M, Ungsunan L, Moschos S, Flier JS, Mantzoros CS (2002). Effects of acute and chronic administration of the melanocortin agonist MTII in mice with diet-induced obesity. Diabetes.

[b38] Rios M, Fan G, Fekete C, Kelly J, Bates B, Kuehn R (2001). Conditional deletion of brain-derived neurotrophic factor in the postnatal brain leads to obesity and hyperactivity. Mol. Endocrinol.

[b39] Rossi G, Lapaczewski P, Diamond MP, Jacob RJ, Shulman GI, Sherwin RS (1993). Inhibitory effect of pregnancy on counterregulatory hormone responses to hypoglycemia in awake rat. Diabetes.

[b40] Ruffin M, Nicolaidis S (1999). Electrical stimulation of the ventromedial hypothalamus enhances both fat utilization and metabolic rate that precede and parallel the inhibition of feeding behavior. Brain Res.

[b41] Ryan EA, O'Sullivan MJ, Skyler JS (1985). Insulin action during pregnancy. Studies with the euglycemic clamp technique. Diabetes.

[b42] Sanz C, Roncero I, Vazquez P, Navas MA, Blazquez E (2007). Effects of glucose and insulin on glucokinase activity in rat hypothalamus. J. Endocrinol.

[b43] Sauve D, Woodside B (1996). The effect of central administration of prolactin on food intake in virgin female rats is dose-dependent, occurs in the absence of ovarian hormones and the latency to onset varies with feeding regimen. Brain Res.

[b44] Unger TJ, Calderon GA, Bradley LC, Sena-Esteves M, Rios M (2007). Selective deletion of Bdnf in the ventromedial and dorsomedial hypothalamus of adult mice results in hyperphagic behavior and obesity. J. Neurosci.

[b45] Vanevski F, Xu B (2013). Molecular and neural bases underlying roles of BDNF in the control of body weight. Front. Neurosci.

[b46] Xu B, Goulding EH, Zang K, Cepoi D, Cone RD, Jones KR (2003). Brain-derived neurotrophic factor regulates energy balance downstream of melanocortin-4 receptor. Nat. Neurosci.

[b47] Zhang Y, Matheny M, Tumer N, Scarpace PJ (2004). Aged-obese rats exhibit robust responses to a melanocortin agonist and antagonist despite leptin resistance. Neurobiol. Aging.

